# Cloning and Expression of a Truncated Form of the p72 Protein of the African Swine Fever Virus (ASFV) for Application in an Efficient Indirect ELISA System

**DOI:** 10.3390/pathogens14060542

**Published:** 2025-05-29

**Authors:** Julieta Sandra Cuevas-Romero, Perla Lucero Zavala-Ocampo, Sonia Pina-Pedrero, Llilianne Ganges, Adriana Muñoz-Aguilera, José Bryan García-Cambrón, Fernando Rodriguez, Aruna Ambagala, José Luis Cerriteño-Sánchez

**Affiliations:** 1 Centro Nacional de Investigación Disciplinaria en Salud Animal e Inocuidad, Instituto Nacional de Investigaciones Forestales, Agrícolas y Pecuarias, Cuajimalpa de Morelos, Mexico City 05110, Mexico; 2 Posgrado en Ciencias de la Producción y de la Salud Animal, Facultad de Medicina Veterinaria, Universidad Nacional Autónoma de México. Av. Universidad 3004, Copilco Universidad, Mexico City 04510, Mexico; plucerozavala@gmail.com; 3 IRTA, Animal Health, Centre de Recerca en Sanitat Animal (CReSA), Campus de la Universitat Autònoma de Barcelona (UAB), 08193 Bellaterra, Spain; sonia.pina@irta.cat (S.P.-P.); llilianne.ganges@irta.cat (L.G.); adriana.munoz@irta.cat (A.M.-A.); fernando.rodriguez@irta.cat (F.R.); 4 Unitat Mixta d’investigació IRTA-UAB en Sanitat Animal, Centre de Recerca en Sanitat Animal (CReSA), Campus de la Universitat Autònoma de Barcelona (UAB), 08193 Bellaterra, Spain; 5 Instituto Colombiano Agropecuario (ICA), Bogotá 110911, Colombia; 6 Posgrado en Biología Experimental, Universidad Autónoma Metropolitana Iztapalapa, Mexico City 09340, Mexico; tlcbioexp@gmail.com; 7 National Centre for Foreign Animal Disease, Winnipeg, MB R3E 3M4, Canada; aruna.ambagala@inspection.gc.ca

**Keywords:** p72, African swine fever, recombinant protein

## Abstract

African swine fever (ASF) is a disease that affects both domestic and wild swine. It was recently reported in the Dominican Republic and Haiti (2021), representing a substantial risk to America. The goal of this study was to produce a truncated form of the ASF-p72 recombinant protein based on the ASF strain genotype II (Georgia 2017) as well as to develop and validate a sensitive and specific ASF indirect-ELISA (iELISA) for early detection of ASF. The truncated ASF-p72 recombinant protein was successfully expressed in *E. coli* BL21/DE3 cells using the pET-SUMO plasmid. Bioinformatics analysis showed 100% homology among the new isolates of ASFV from genotype II. The ASF-p72-truncated protein was used to develop an iELISA, which had a high sensitivity (88%) and strong specificity (97%); the concordance index kappa was K = 0.872, indicating nearly perfect agreement compared to the WOAH confirmatory immunoperoxidase test. The validation results utilizing the reference sera panel from the OIE-ASF Reference Laboratory show the excellent detection capabilities of ASF antibodies up to a 1:1000 serum dilution. The inter-assay coefficient of variation (CV 10.4%) and intra-assay CV (2.8%) data show that the assay is precise and reproducible. This biotechnology advancement can be used to conduct future epidemiological research for ASF surveillance in ASF-free American countries.

## 1. Introduction

African swine fever (ASF) is a disease that affects both domestic and wild swine and has spread across Central Europe, East and Southeast Asia, and Africa. Currently, ASF is present in more than 20 sub-Saharan African countries [1]. Moreover, since the virus appeared in Georgia, a part of the Caucasus region, in 2007 [2], ASF has extended its geographical distribution and is currently present in large parts of Eastern, Central, and Southern Europe. Furthermore, in 2018, ASF was introduced into China and has since spread widely in the region [3]. After more than 40 years of being absent in the Western Hemisphere, the virus made the long-distance jump to appear in the Dominican Republic and Haiti in 2021. The 2021 outbreak in the Dominican Republic was caused by an isolate that has been identified as a derivative of the pandemic strain currently circulating in Asia and Europe, namely, genotype II ASFV5 [4,5]. This fact poses a significant risk to the rest of the Americas and places a significant burden on health authorities to implement appropriate measures to avoid the spread of ASF to other nations in the Americas.

ASF is caused by the ASF virus (ASFV), a double-stranded DNA virus with a complex molecular structure. It is the only member of the Asfarviridae family [6] and the only DNA virus transmitted by arthropods, as it is carried by soft ticks of the Ornithodoros genus [7,8]. The ASFV genome consists of a double-stranded DNA virus and varies from 170 to 194 kb in size. It encodes 150–200 viral proteins, including 68 structural proteins and more than 100 non-structural proteins. At least 16 of the 68 detected ASFV proteins are thought to be involved in the assembly of the virus particle, 26 virus-encoded proteins have predicted transmembrane domains, and at least two viral proteins (pA224L and pEP402R) might interfere with host defense mechanisms [9,10]. p72 is the major capsid surface protein that is used for serotyping ASFV strains due to its conserved character, and it mediates the adsorption of ASFV [10]. In addition, due to the stability of the p72 gene in some field isolates from different outbreaks, a recent study classified ASFV into six genotypes based on p72 amino acid sequences [11]. Packaging of the major envelope protein p72 requires the assistance of a molecular chaperone encoded by B602L. Inhibition of pB602L synthesis decreases the p72 protein. Binding of pEl02R and pE120R to the major envelope protein p72 also has an essential effect on virus transport from the assembly site to the cytomembrane. ASFV protein 72 (p72) is a major structural protein that contains a conformational neutralizing epitope and elicits robust immune responses, which serve as the foundation for the majority of modern serological techniques [10,12,13].

After 8 days of ASFV infection, the antibody level gradually increases, suggesting a humoral immune response in the host. These neutralizing antibodies have a certain protective effect. In addition, cellular immunity, such as with CD8a+ T cells, also plays an important antiviral role in ASFV infection. CD4 + CD8 + double positive (DP) T cells can secrete perforin and granzyme, which may also play a role in resisting ASFV infection [14]. Infection by ASFV causes apoptosis in lymphocytes and associated lymphopenia, which influences the immune response capacity against the virus [10,15].

Several African swine fever virus proteins have been reported to induce neutralizing antibodies in immunized pigs, and vaccination strategies based on DNA vaccines and recombinant proteins have also been explored; however, these have not been very successful. The complexity of the virus particle and the ability of the virus to modulate host immune responses are the most likely reasons for this failure [16,17]. The rapid and reliable detection of African swine fever virus (ASFV) is essential, both for the timely implementation of control measures to prevent the spread of the disease and to differentiate African swine fever (ASF) from other pig diseases with similar clinical presentations [18]. Prevention and control efforts, therefore, rely primarily on rapid and reliable laboratory diagnostics and strict sanitary measures. Extensive studies have been carried out to assess the specificity and sensitivity of ASF serological tests.

Currently, the World Organization for Animal Health (OIE) uses a combination of serological and antigen-detection tests to diagnose African swine fever (ASF). These tests include enzyme-linked immunosorbent assay (ELISA), immunoblotting (IB), and indirect immunofluorescence (IIF). The indirect immunoperoxidase test (IPT), validated by the European Union Reference Laboratory (EURL) for ASF, has effective analytical and diagnostic sensitivity and can be used as an alternative confirmatory test for the diagnosis of ASF using porcine sera [2,19].

The most commonly used test is the ELISA [2,20], which is suitable for examining serum or plasma. The disease caused by this virus can manifest in various forms, including peracute, acute, subacute, or chronic, where the presence of anti-ASFV antibodies suggests infection. Furthermore, anti-ASFV antibodies emerge shortly after infection (7–10 days) and can last for months or even years. Domestic pigs and wild boars infected with virulent strains typically die before a specific antibody immune response develops. In places with a well-established ASFV infection, where attenuated and low-virulent virus isolates are also present, serological detection is critical for detecting recovered and asymptomatic infected animals [16,18,20].

In recent years, extensive studies have been carried out to assess the specificity and sensitivity of ASF serological tests under the different epidemiological scenarios of Africa and Europe [17,21]. Despite the lack of neutralization, humoral immunity is vital in defending against ASFV, delaying illness onset, and even protecting against repeated infections, as established by a passive antibody transfer experiment [22]. The lack of entirely neutralizing antibodies has been one of the most significant challenges in developing an effective vaccine.

The overall goal of this study was to produce an overexpression of truncated p72 recombinant antigen of ASFV in a low-cost *E. coli* vector system. This antigen can be utilized to create serological techniques such as indirect ELISA (iELISA), which detects antibodies in a sensitive and specific assay.

## 2. Materials and Methods

### 2.1. Reference Swine Serum Samples

A total of 69 pig sera samples were used to standardize and validate iELISA based on the ASF-p72-truncated protein. A total of 46 negative serum samples from clinically healthy swine farms and 23 positive sera from naturally infected pigs of the past ASFV epizootic in Spain in the 1990s (ASFV genotype I) were used. The collection of sera controls was provided by the Institute for Research and Technology in Food and Agriculture (IRTA), Programa de Sanitat Animal, Centre de Recerca en Sanitat Animal (CReSA), (IRTA-CReSa), Barcelona, Spain (Supplementary Tables S1 and S2). These control sera were confirmed as true negatives and positives by the immunoperoxidase test (IPT), which is a confirmatory antibody detection test for ASF (WOAH, 2022, OIE) [19]. All clinical serum samples were previously tested by the reference IPT and the commercial ASFV-blocking ELISA antibody detection kit (Ingenasa-Ingezim PPA Compac K3; Ingenasa, Madrid, Spain), which detects the ASFV p72 antibody. Furthermore, a validation was performed with different reference panel sera (2019, 2020, 2021, and 2022) of the interlaboratory intercomparison serological assay for ASF (Ingenasa-Ingezim PPA Compac K3; Ingenasa, Madrid, Spain), which uses a monoclonal antibody (MAb) specific to VP72 ASFV protein) from the OIE Reference Laboratory “Laboratorio Central de Veterinaria”, Algete, Madrid, Spain. Fourteen positive serum samples from experimentally ASFV-infected pigs with cepa NHV/68 were analyzed at various dilutions, and five sera from uninfected pigs were included.

### 2.2. In Silico and Bioinformatics Analysis of ASF-p72-Truncated Protein

Different algorithms, introduced by Jameson-Wolf [23] and Kolaskar and Tongaonkar [24], were used to predict potential antigenic sites and select the appropriate protein region for expression in the *E. coli* vector system. The sequence of the ASF-p72-truncated protein was analyzed using bioinformatics programs. The Kyte and Doolittle (1982) [25] algorithm was used to predict the antigenic index, hydrophilicity, and surface probability along the amino acid sequence in a given region on the surface of the protein, which was assessed using the approach of Emini et al. (1985) [26]. Glycosylated sites were predicted using NetNGlyc 1.0 [27]. Finally, the prediction structure of the molecular 3-D model of the ASF-p72-truncated protein and epitopes was obtained using UCSF Chimera software 1.18 (University of California, San Francisco, CA, USA), with the major capsid protein p72 structure of the African swine fever virus (PDB accession: 6KU9) used as a template [13]. In addition, to determine the conservation of the ASF-p72-truncated protein, multiple sequence alignment comparisons were performed using Georgia 2007/1 as a reference strain and including 35 sequences (each composed of 254 amino acids) from old and new isolates of different ASFV representative genotypes, such as Dominican Republic/1996/1980 and Brazil p72 (B646L), using the MEGA X program and the Clustal W algorithm. Phylogenetic trees were obtained using the maximum likelihood based on the JTT model and gamma distribution.

### 2.3. Cloning, Subcloning, and Overexpression of ASF-p72-Truncated Protein

The p72 full gene (B646L) was amplified by PCR from the ASF Georgia reference strain 2007 (GenBank Accession No. FR682468) using specific primers: Fw 5′-ATGGTAGGTCTCAAATGATGGCATCAGGAGGAGCTTTTTG-3′ (forward) and Rv 5′ATGGTAGGTCTCAGCGCTGGTACTGTAACGCAGCACAGCT 3′ (reverse). The resulting products were cloned into backup vectors pASK-IBA33 plus plasmid (IBA US Distribution Center 2-1433-000) on *Escherichia coli* (*E. coli*) strain TOP10 cells at the Institute of Agrifood Research and Technology, Centre de Recerca en Sanitat Animal in Barcelona, Spain. The cloned products were sequenced by Sanger technology, and the sequences were analyzed using the DNAstar bioinformatics package to construct expression plasmids for the truncated protein region (762 bp). The open reading frame (ORF) of a fragment comprising residues 73 to 326 aa was amplified using specific primers: Fw 5′-GAA TAC AAT AAA GTA CGC CCG CAT ACG-3′ and Rv 5′-CTA CTA AGG GGG CTG ATA GTA TTT AGG GGT-3′. Cloning and expression were performed according to the procedures described by Lara et al. [28]. Briefly, temperature-gradient end-point PCR was used to select the best PCR conditions with cDNA, and the PCR product was subcloned into the Champion™ pET-SUMO expression vector (Thermo Fisher Scientific, Waltham, MA, USA) and verified by nucleotide sequencing with Sanger technology at the Biotechnology Institute of UNAM. Competent cells of *E. coli* strain One Shot™ BL21 (DE3) (Invitrogen, Carlsbad, CA, USA) were used to obtain the overproduction strain (BL21-p72-truncated protein). The obtained recombinant plasmids in *E. coli* were purified according to Castañeda-Montes et al. [29]. To express BL21-p72-truncated protein, 200 mL of LB plus kanamycin (50 µg/mL) medium was inoculated with a pre-culture (200 µL of LB + kanamycin [50 μg/mL]) and incubated at 37 °C at 250 rpm for 2 h. Then, isopropyl β-D-1-thiogalactopyranoside (IPTG) 1.5 mm (final concentration) was added to express the protein. After 12 h of induction, recombinant p72-truncated protein was extracted from the inclusion bodies by solubilizing it in N-lauroylsarcosine (9%) and 50 mM Tris-HCl (pH 8) and purifying it using a 5 mL HisTrap^®^ Chelating High-Performance Ni-NTA agarose column (GE Healthcare, Chicago, IL, USA). This was followed by SDS-PAGE staining and Western blot using a 6x-His monoclonal antibody (Invitrogen, Rockford, IL, USA) to confirm the expressed protein. Protein concentration was determined using the Bradford assay.

### 2.4. Antigenic Evaluation of ASF-p72-Truncated Protein in a Mouse Model

The humoral immune response was evaluated in mice to determine the antigenicity of the ASF-p72-truncated protein (ASF-p72t). Briefly, 3-week-old CF-1 mice were divided into three groups (n = 8 per group). The mice were immunized via subcutaneous (SC) administration of 200 μL doses, formulated with 200 ng of protein per mouse, which were injected into a fold of skin in the neck, with boosters administered after 2 weeks. The immunization and bleeding scheme were as follows: Group 1 was immunized with ASF-p72-truncated protein mixed with Matrix-M^TM^ adjuvant (Isconova AB, Uppsala, Sweden) (ASF-p72t + Matrix-M^TM^); Group 2 was immunized with the ASF-p72-truncated protein plus PBS (ASF-p72t + PBS); and Group 3 was injected with PBS as a blank control group. Blood samples were collected from the tail vein at days 0, 7, 14, 21, 28, and 35 post-immunizations for antibody detection by iELISA, as previously described [30], with minor modifications, such as using ASF-p72-truncated protein as antigen in a concentration of 75 ng/well on a coated plate. Statistical analyses of the results were performed using two-way ANOVA and a post hoc Dunnett’s multiple comparison test to compare groups immunized with protein plus adjuvant versus protein alone on different days. Differences at *p* < 0.05 were considered statistically significant with a 95% confidence interval (* *p* < 0.05; ** *p* < 0.005; *** *p* < 0.0005), and graphs were constructed using SigmaPlot version 12.5 (Systat Software Inc., San Jose, CA, USA). All procedures were in accordance with Mexican legislation (NOM-062-ZOO-1999; SAGARPA) [31], based on the Guide for the Care and Use of Laboratory Animals, NRC. The experiment was previously approved under a permit from CBCURAE-2017/001 (Institutional Animal Care and Use Committee), CENID-SAI, INIFAP.

### 2.5. Standardization of Indirect ELISA (iELISA) of ASF-p72-Truncated Protein with Pig Serum Samples

The iELISA assay conditions were determined using checkerboard titration to find the optimal antigen coating concentrations and serum dilutions. Briefly, ASF-p72-truncated protein was double diluted from 1:125 ng to 1:15 ng per well in 50 mM carbonate-bicarbonate buffer (pH = 9.6; 0.5 M Na_2_CO_3_ and 0.5 M NaHCO_3_) and coated onto MaxiSorpTM 96-well microplates (Nalge Nunc International Corp., Rochester, NY, USA) with a volume of 100 μL/well to be absorbed overnight at 4 °C. Subsequently, plates were washed once, blocked with 5% skimmed milk, and incubated at 37 °C for 1.5 h. ASFV-positive and ASFV-negative control sera were diluted (1:50, 1:75, 1:100, and 1:200), added to the plate in a volume of 100 μL/well, and incubated at 37 °C for 1 h. After washing four times with PBS-Tween, 0.1%, horseradish peroxidase (HRP)-labeled anti-pig-IgG (Sigma, St. Louis, MI, USA) was added as the secondary antibody at ratios of 1:15,000, 1:20,000, and 1:22,500 and placed in a volume of 100 μL for 1 h at 37 °C to obtain the best reaction conditions. Following five rounds of washing, 100 μL of 3,3′,5,5′-tetramethylbenzidine (TMB) chromogenic solution (SeraCare, Milford, MA, USA) was applied to each well and incubated at ambient temperature for 13 min while being protected from light. Finally, the reaction was stopped with 100 μL of 2 M sulfuric acid, and the absorbance was measured at an optical density of 450 nm (OD_450nm_) [32].

The optimized iELISA was repeated ten times using a selected ASF-strong positive serum sample and evaluated with the IPT assay according to the OIE Validation Guideline 3.6.1 [19], with a high IPT antibody index of 1:1280 named as strong positive (C++) and an IPT antibody index of 1:180 named as weak positive (C+), from naturally infected pigs collected during the past ASFV epizootic in Spain (1990s); a negative control serum sample (C−) was also included. The results of the iELISA were expressed by analyzing the optical density readings (OD_450nm_) and calculating the coefficient of variation (CV). To assess assay reproducibility, the coefficient of variation (CV) was calculated, considering the inter-assay CV as an expression of plate-to-plate consistency, calculated from the mean values for the high (C++) and low controls (C−) on each plate, where inter-assay CVs of less than 15% were generally deemed acceptable. These were calculated as follows [33,34]:

Inter-assay % CV (n = 10) = average of strong positive control CV + average negative control CV/2 × 100

For the intra-assay analysis reported in this study, each serum sample was run in duplicate on coated plates. The percent CV for each sample was calculated by finding the standard deviation of results 1 and 2, dividing by the average, and multiplying by 100. The average of the individual percent CVs was reported as the intra-assay percent CV. Intra-assay percent CVs should be less than 10% to reflect the performance of the assay. This was calculated as follows:

Intra-assay % CV (n = 43) = average % CV

### 2.6. Statistical Analysis (Sensitivity, Specificity, and Index Kappa) of iELISA

The specificity, sensitivity, and index kappa (κ) of iELISA were calculated using the Win Episcope program [35]. Sensitivity and specificity values were determined by comparing the tested reference control sera (true positive and true negative) with the IPT results using a 2 × 2 contingency table. Percentages of sensitivity and specificity were calculated as follows:

% sensitivity = [a/(a + c)] × 100; % specificity = [d/(b + d)] × 100

In the equation, a is the number of true positives, b is the number of false positives, c is the number of false negatives, and d is the number of true negatives.

The agreements of both tests were calculated as the kappa index (κ), which represents the proportion of observed agreements beyond chance in relation to the maximum possible agreement beyond chance, considering the IPT assay as the reference test (“Gold Standard”).

κ = (Po − Pe)/(1 − Pe)

where Po = (a + d)/N; Pe = (rt + su)/N^2^; r = a + b; t = a + b; s = c + d; u = b + d; N = a + b + c + d.

The interpretation of the kappa value (κ) was determined, with 0.81 representing almost perfect agreement, values of 0.61 to 0.80 representing substantial agreement, values of 0.41 to 0.60 representing good agreement, values of 0.21 to 0.40 representing moderate agreement, values of 0.01 to 0.20 representing slight agreement, and values of 0.00 representing no agreement [36,37].

During the validation process, serum samples that were false negative or positive were confirmed by the IPT test according to the protocol established by the OIE Reference Laboratory [19].

### 2.7. Determination of the iELISA Cut-Off Point

The cut-off point was set at the level of antibody in serum that distinguishes between positive and negative animals [38]. Identifying the cut-off point is critical in every serological experiment. To determine the cut-off, the sera controls confirmed as real negatives (n = 46) and positives (n = 23) by the immunoperoxidase test were evaluated by standardized iELISA. The average value ($\bar{X}$) of OD_450nm_ of negative sera plus two standard deviations (SDs) was defined as the cut-off value, with a final critical value of $\bar{X}$ + 3SD. Serum specimens with OD_450nm_ greater than or equal to the critical value were deemed ASF positive. Sera with an optical density between the cut-off point and the critical value were considered inconclusive, and the result was confirmed by IPT; if not, they were considered negative.

## 3. Results

### 3.1. In Silico Analysis of ASF-p72-Truncated Protein

**Figure 1:**
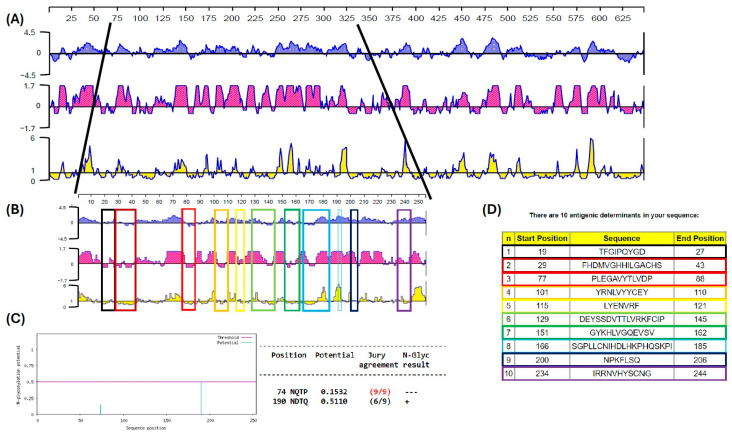
(**A**) Amino acid sequence of African swine fever p72 protein (B646L) from the Georgia 2007 sequence (646 aa residues). (**B**) Amino acid sequence of a fragment selected as the best candidate for the expression of ASF-p72-truncated protein (73-254 aa residues). Hydrophobicity, antigenicity index, and surface probability are represented by the Kyte and Doolittle hydrophobicity plot (blue: hydrophobic regions), Jameson–Wolf antigenic index (pink), and Emini surface accessibility prediction (yellow); red boxes indicate protein sequence regions where the antigenic determinants were found using the Jameson and Wolf analysis (n = 10). (**C**) Prediction of two glycosylated sites in the protein fragment at 190 aa residue (blue lines). (**D**) Prediction of antigenic determinant in the ASF-p72-truncated protein (the same antigenic regions are marked in (**B**) with different colors).

In silico analyses were carried out with African swine fever isolated from Georgia in 2007 (accession number: FR682468). As a result, a protein fragment with 254 amino acid (aa) residues and a weight of 28,651 kDa was designated as an antigenic protein that contains the most immunogenic epitopes and best candidates for cloning and expression in the pET SUMO vector. Analysis showed segments with high antigenicity indexes throughout the sequence, represented by positive values in the diagrams shown in Figure 1A. The ASF-p72-truncated protein comprising residues 73 to 326 aa, beginning with glutamic acid (Glu, E) at residue 73 and ending with proline (Pro, P) at residue 326, was selected from the N-terminal region. From the logarithms analysis, high hydrophobicity sites were detected in the protein region, with the highest antigenicity index throughout the sequence. The surface probability, hydrophilic regions, and two glycosylated regions are shown in Figure 1B,C. The presence of three significantly higher peaks in the surface probability plot suggests that there are three regions in the protein with a higher probability of being exposed on the surface, which allows greater solubility of the protein.

**Figure 2:**
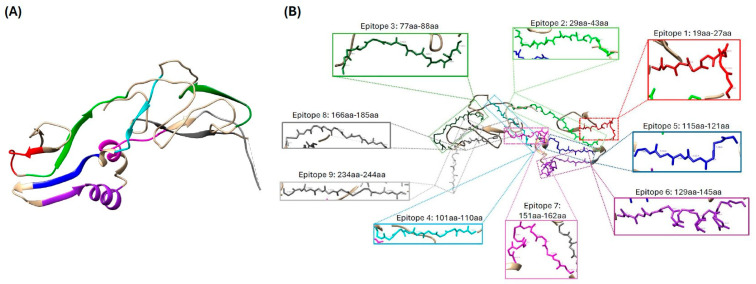
(**A**) A proposed model of structural analysis. (**B**) Nine epitopes localization of ASF-p72-truncated protein.

According to the antigenic analysis of the ASF-p72-truncated protein, ten antigenic determinants (epitopes) were predicted by Jameson and Wolf, 1988 [23], as potential regions that can be recognized by immune cells, nine of which are exposed on the surface of the 3-D model structure of the protein and correspond to the contact surface and hydrophilic areas that can potentially induce an immune response (Figure 1D and Figure 2A,B).

### 3.2. Bioinformatics Analysis of ASF-p72-Truncated Protein

**Figure 3:**
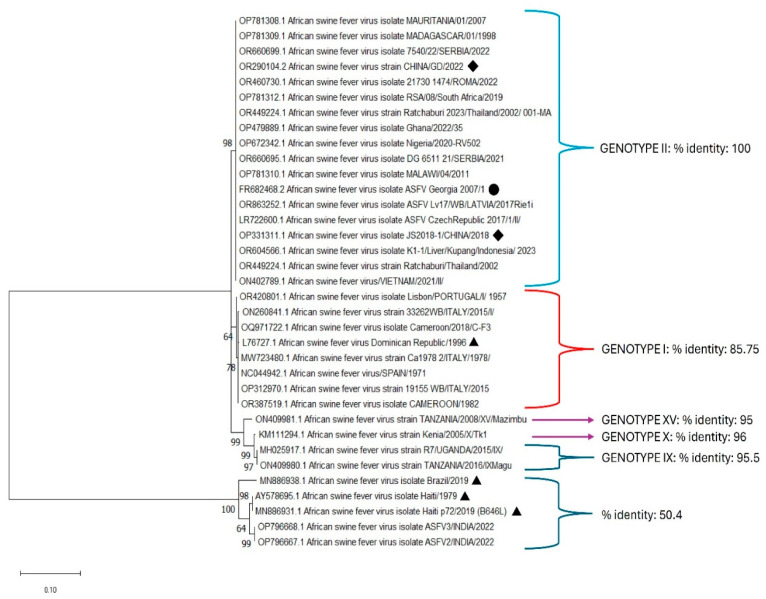
Phylogenetic trees were created using the MEGA X software, with at least 1000 replicates, using the maximum likelihood based on the JTT model and gamma distribution. ▲ Strains reported in America; ● Reference strain Georgia 2007; ◆ Strains reported in China.

Multiple sequence alignment of 35 homologous nucleotide and amino acid sequences of the ASF-p72-truncated protein showed conservation and perfect agreement with the sequence of genotype II (100% identity), including Georgia 2007/1 and China 2018/2022. Phylogenetic trees were created using the MEGA X software, version 10.2.6, and evolutionary analysis using the maximum likelihood method was inferred using the JTT matrix-based model. The bootstrap consensus tree inferred from 1000 replicates was taken to represent the evolutionary history of the taxa analyzed. The initial tree for the heuristic search was obtained automatically by applying Neighbor-Join and Bio NJ. This analysis involved a total of 241 positions in the final dataset (Figure 3).

### 3.3. Cloning and Expression of ASF-p72-Truncated Protein

**Figure 4:**
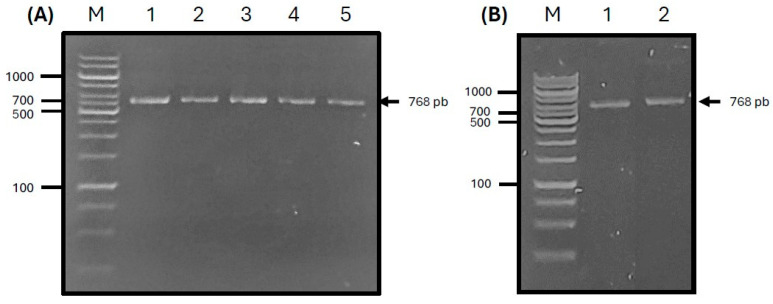
(**A**) PCR amplification of a fragment from the B646L gene by temperature gradient PCR. M: base pair marker 100 bp; lane 1: 63.5 °C; lane 2: 64.3 °C; lane 3: 67.6 °C; lane 4: 68 °C; lane 5: 71.7 °C. (**B**) pET-SUMO-ASF-p72-truncated protein plasmid. For the PCR reaction conditions, 68 °C is used. M: base pair marker 1 kb; lane 1/2: recombinant plasmid.

cDNA from the ORF of the ASF-p72 gene (pB602L) was amplified from the 2007 ASF Georgia reference strain (GenBank Accession No. FR682468). The results of PCR products were observed at the expected molecular weights (e.g., 1938 bp). After correct sequence analysis, the PCR amplicon was cloned into the pASK-IBA33 vector using *E. coli* Top10 bacterial cells (Figure 4A), which was then cloned into the pET-SUMO expression vector to generate a recombinant plasmid (pET-SUMO-ASF-p72-truncated protein) (Figure 4B) and transferred into *E. coli* BL21 (DE3) recipient cells to express the protein.

**Figure 5:**
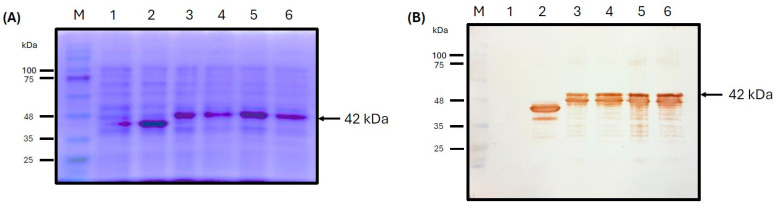
ASF-p72-truncated protein was expressed as inclusion bodies. Coomassie stain (**A**) and Western blot (**B**) notably show the protein in greater quantity, making it easy to identify. The considerable signal observed is indicative of the efficiency of the expression of recombinant protein at ~42 kDa expected molecular weight (arrow on lanes 3, 4, 5, and 6). M: marker; lane 1: negative control; lane 2: positive control (CAT protein/37 kDa).

The recombinant ASF-p72-truncated protein was successfully expressed in the *E. coli* BL21 (DE3). SDS-PAGE analysis revealed an overproduced band of recombinant protein recovered from an insoluble phase (inclusion body) at the expected molecular weight (42 kDa) in four samples of bacteria transformed with the pET-SUMO-ASF-p72-truncated protein plasmid (Figure 5A). This was confirmed by Western blot detection (Figure 5B) using a specific anti-histidine monoclonal antibody present in the C-terminal of the recombinant protein. Qualitatively, a large amount of protein was expressed compared with non-induced cultures that did not express the protein.

**Figure 6:**
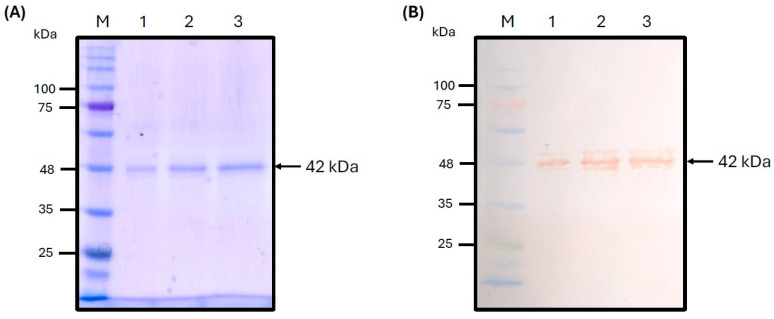
Purified ASF-p72-truncated recombinant protein (ASF-p72t) from inclusion bodies by IMAC. Coomassie stain (**A**) and Western blot (**B**). (M: marker; lane 1: 5 µL of ASF-p72t; lane 2: 10 µL of ASF-p72t; lane 3: 15 µL of ASF-p72t).

The ASF-p72-truncated protein was purified using Ni-NTA agarose by immobilized metal affinity chromatography (IMAC); the elution phases showed a specific signal of the expected molecular weight of ~42 kDa, obtained by one step of affinity chromatography; it was visualized by SDS-PAGE and Western blot (Figure 6). A linear regression was performed to quantify the concentration of the purified ASF-p72-truncated protein. The equation of the straight line for the regression was y = 0.013x + 0.197, with R2 = 0.9719. Finally, the average yield of these one-step purified proteins for the ASF-p72-truncated protein was 41 μg/mL of purified recombinant protein with a yield of 231 μg/100 mL of culture.

### 3.4. Antigenicity of ASF-p72-Truncated Protein by Mice Immunization

**Figure 7:**
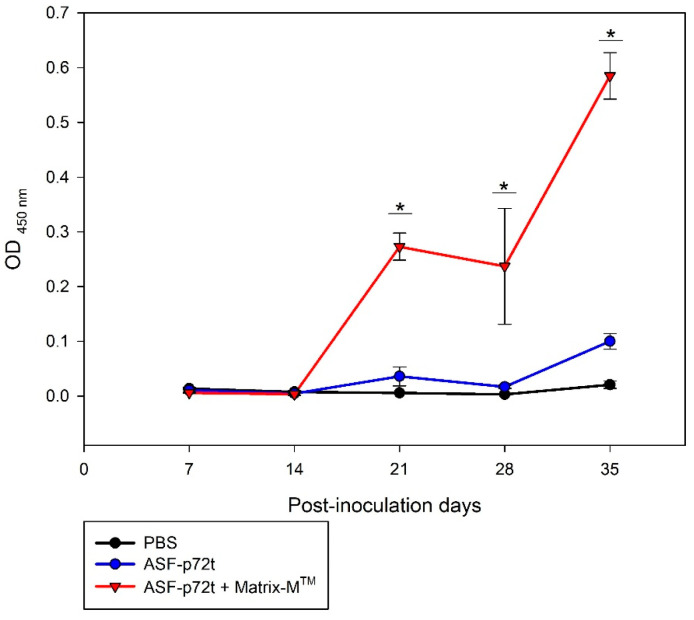
Graph shows humoral immune response in mice immunized with ASF-p72-truncated recombinant protein (ASF-p72t) by iELISA. * *p* < 0.05.

The antigenic capacity of the ASF-p72-truncated protein in vaccinated CF-1 mice was evaluated using data on the kinetics of antibody production detected by iELISA (Figure 7). A high level of antibody production specific to ASF-p72-truncated protein plus adjuvant Matrix-M^TM^ was detected from day 14 post-inoculation, with an increase in levels at day 28 that was maintained until day 35. The antibody detection in mice immunized with recombinant ASF-p72-truncated protein plus PBS showed a slight immune response starting on day 28 post-inoculation, with an increased level of antibody at day 35.

### 3.5. Standardization and Validation of Indirect ELISA (iELISA) of ASF-p72-Truncated Protein

The optimal coating conditions were determined using the “block titration” model, which indicated a concentration of 50 µg/well of p72 recombinant protein diluted in carbonate buffer (0.05 mol/L, pH 9.6) and incubated overnight at 4 °C, followed by blocking with 5% skimmed milk powder for 1.5 h at 37 °C. The optimal incubation duration for serum was 1 h at 37 °C at a 1:100 dilution and 1 h at 37 °C for enzyme-labeled secondary antibody with anti-Pig-HRP. The optimal conjugate dilution (anti-Pig-HRP) was titrated using the reference control sera at a dilution of 1:25,000, where the substrate reaction kinetics determined the greatest difference between positive and negative sera, and the optimum color development time was 12 min following the reaction. To assess the reproducibility of the assays, the standardized iELISA was repeated 10 times with ASFV-positive and ASFV-negative control serum at BSL2 and BSL3, and the results are shown in Table 1. The frequency distribution of positive or negative sera was determined in the optical density ranges, plus two standard deviations (2 SD), with the following results: for the strong positive control serum (C++), the range was 0.786 to 1.50 OD; for the weak positive control serum (C+), the range was 0.326 to 0.596 OD; and for the negative control serum (C−), the range was 0.08 to 0.208 OD. The data points were relatively close to the mean, suggesting the stability and consistency of the standardized iELISA with high reproducibility inter-assay with CV = 10.4% (Table 1).

According to the standardized iELISA, the validation results using 14 positive serum samples from the reference sera panel of the OIE-ASF Reference Laboratory were obtained (Supplementary Table S1).

**Figure 8:**
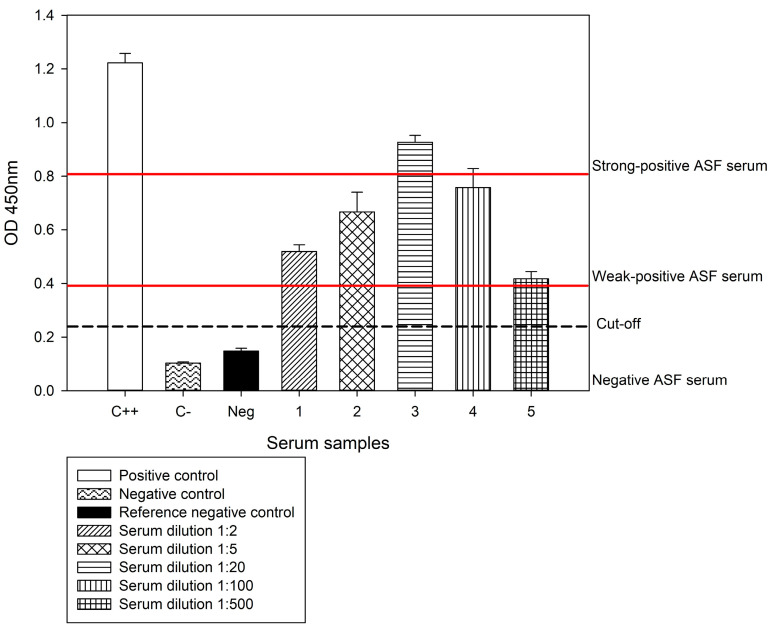
High antibody detection capacity of the ASF-p72-truncated iELISA of serum dilution samples from the IRTA-CReSA OIE Reference Laboratory sera panel (Table 2).

The results indicated an average OD of 0.659 and a standard deviation of 0.198, classifying all positive samples as weak positives. This demonstrates the ASF-p72-truncated protein assay’s excellent detection capacity, which can identify samples up to 1:500 plus the dilution factor equivalent to 1:1000 (Table 2; Figure 8).

### 3.6. Determination of Sensitivity, Specificity, and Concordance of iELISA

The sensitivity and specificity of the assay were calculated using a 2 × 2 contingency table; under these experimental conditions, a comparative sensitivity of 88% and a comparative specificity of 97.72% were calculated. The concordance index kappa (κ) of iELISA was κ = 0.872, indicating a nearly perfect agreement by considering IPT as the “gold standard” (Table 3).

Here, Po is the proportion of agreements observed, Pe is the proportion of agreements expected in the hypothesis of independence, r is the sum value of a + b, s is the sum value of c + d, t is the sum value of a + c, u is the sum value of b + d, and N is the total (a + b + c + d).

**Figure 9:**
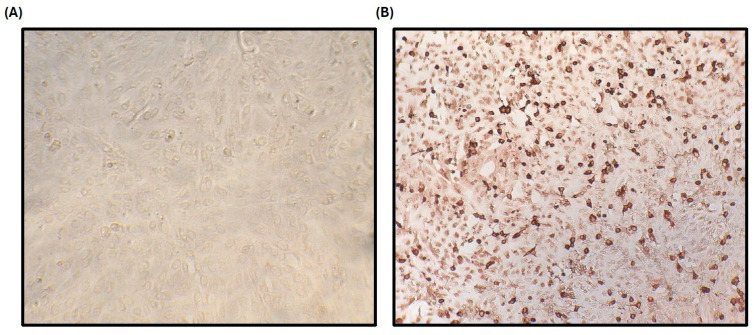
Indirect immunoperoxidase test (IPT) confirmed the true negative (**A**) and positive (**B**) pig serum samples.

In addition, the serum samples that resulted in false negatives or positives were confirmed by the IPT test as confirmatory antibody test detection for ASF (Figure 9).

### 3.7. Determination of Cut-Off Values

**Figure 10:**
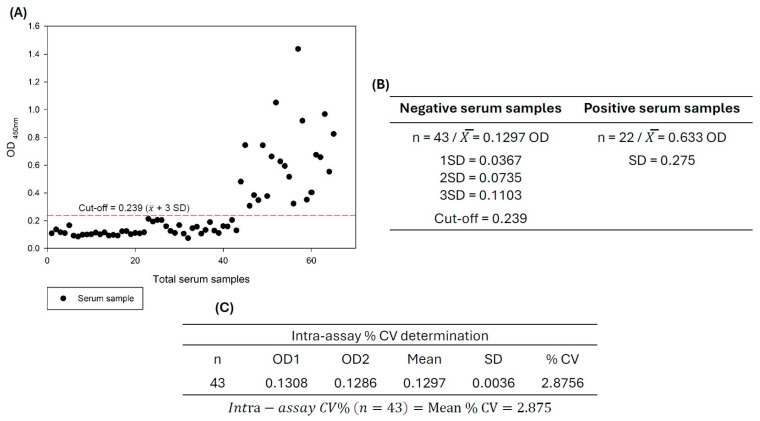
Serological distribution of positive and negative serum samples (**A**) tested by iELISA of ASF-p72-truncated protein. The dotted line represents the cut-off value for iELISA and was calculated as 0.239, testing 43/46 negative serum samples and 22/23 positive serum samples (**B**). Sera above the dotted line were considered ASF positive, and sera below the dotted line were considered ASF negative according to the cut-off value. Intra-assay (% CV) determination (**C**) using 43 negative sera.

The cut-off value was determined by testing 43 negative reference sera using optimized iELISA conditions, resulting in an $\bar{X}$ of 0.129 OD with a standard deviation (SD) of 0.036. The established cut-off value was 0.203 ($\bar{X}$ + 2 SD) with a final critical value of $\bar{X}$ + 3 SD = 0.239 (Figure 10). Serum specimens with an optical density higher than the cut-off point were deemed positive, sera with an optical density below the cut-off point were considered negative, and sera with an optical density between the cut-off point and critical value ($\bar{X}$ + 3 SD = 0.239 OD) were considered inconclusive; the result needs to be confirmed by IPT.

The frequency distribution of positive ASF sera samples from naturally infected pigs (n = 22) was determined in the OD range of 0.347 to 0.919 ($\bar{X}$ ± 1 SD), where the lowest value (0.347) of the optical density units represents 30% of the positivity percentage (%PP) in relation to the average OD value of the strong positive serum control (C++ 1.143 OD); for the negative sera samples (n = 43), the OD range was 0.076 to 0.239 ($\bar{X}$ + 3 SD), where the highest value of the negative serum corresponds to 20% PP values in relation to C++ (1.143 OD). The intra-assay evaluation was calculated by comparing duplicates for 43 samples (Supplementary Table S2). The % CV for each sample was calculated by finding the standard deviation of results OD 1 and OD 2, dividing that by the duplicate mean, and multiplying by 100. The average of the individual CVs was reported as the intra-assay CV of 2.8%, where CV < 10%; this score reflects the good performance of the assay.

## 4. Discussion

African swine fever (ASF) is a disease seen in domestic and wild swine that has spread throughout a large geographical area and generally causes high mortality and substantial household financial losses. Recently, ASFV was detected in the Dominican Republic and Haiti 2021 [39]. It was caused by an isolate that has been determined to be a derivative of the pandemic strain currently circulating in Asia and Europe, related to a genotype II of ASFV [40]. Therefore, this work aimed to produce a recombinant ASF-p72-truncated protein based on the ASF Georgia 2017 p72 protein belonging to ASF strain genotype II to develop and validate a sensitive and specific ASF iELISA assay. Early detection, diagnosis, and prevention are crucial in preventing and managing ASF, and serological assays, especially ELISA, are critical for surveillance to regain freedom after a potential ASF outbreak [4].

The bioinformatics analysis of multiple sequence alignment of the recombinant truncated ASF-p72 protein compared with ASFV published sequences showed 100% homology among most of the new isolates of ASFV reported in the gene bank from genotype II, representing African, European, and Caribbean isolates, and the conservation of important residues in the active sites of the truncated ASF-p72 protein [11,41]. This suggests that the expression of the antigenic recombinant protein could be a good candidate to develop serological tools such as indirect ELISA for high-throughput antibody detection derived from the Georgia 2017 sequence.

Furthermore, three-dimensional modeling analysis and antigenic predictions showed that the conformational and functional domains of the recombinant truncated ASF-p72 protein were preserved. Nine B cell epitopes in surface regions of the ASF-p72-truncated protein, which covers over 90% of the protein sequence, demonstrated high antigenicity indexes. This finding agrees with the results of Miao et al., 2023 [42], where the epitopes of the ASF-p72-truncated protein matched in a sequence range of 70 to 300 aa, indicating a region with properties that allow for the formation of a suitable immune response and implying that the ASF-p72-truncated protein could be used as a candidate antigen. These results were further confirmed with functional assays demonstrating that this protein maintained antigenicity and immunogenicity characteristics like native protein. This is important because the ASF-p72 protein is the major capsid protein and the most dominant structural component of the virion [43], making it one of the major antigens detected in infected pigs [13].

The rapid and reliable detection of ASFV is essential, both for timely implementation of control measures to prevent the spread of disease and to differentiate ASF from other pig diseases with similar clinical presentations [19]. Serological diagnosis of ASF is critical for identifying asymptomatically infected animals and those that have recovered, as antibodies to ASF typically appear 7–10 days post-infection and can persist for months or even years [44]. ELISA is a standard serological test that can be used for large-scale testing and is also a designated experiment specified by OIE for international trade to detect specific antibodies to ASFV [4,45]. Nowadays, all commercial ASFV ELISA kits are produced outside Mexico, and their high cost would make them challenging to use for country-wide serological surveys in Mexico.

The ASF-p72-truncated protein was used to develop an ELISA for detecting ASFV antibodies in infected pigs, resulting in a more precise diagnosis with high diagnostic sensitivity (88%) and strong specificity (97%) compared with the specificity of two of the currently available commercial ELISA kits. For example, blocking Ingenasa-ELISA, based on the use of monoclonal antibodies against the P72 ASFV protein (Ingezim PPA Compac K3; Gold Standard Diagnostics, Madrid, Spain), shows 84.3% specificity, and indirect Svanova-ELISA, based on recombinant P30 ASFV proteins as the antigen (Svanovir ASFV-Ab; Boehringer Ingelheim Svanova, Uppsala, Sweden), shows 91.4% specificity. On the other hand, the multiantigen indirect ELISA kit (IDvet-ELISA) for the detection of antibodies against P32, P62, and P72 ASFV proteins (ID Screen African swine fever indirect assay; Grabels, France) has been reported to have 100% specificity [19]. This suggests that using more than two antigens to detect the target antibody of ASF could help with serological diagnosis [46,47]. Furthermore, this assertion contradicts recent studies on the development of a commercial indirect ELISA (AsurDx^TM^) formed by two recombinant proteins, p54 and p72, which showed that the sensitivity was reduced by 83.72% compared to the ASF-p72-truncated protein iELISA (88%). This decrease in sensitivity, even when two proteins are used, contradicts advances in multi-antigen indirect ELISA kits, which use more than two antigens to perform well in detection and discrimination of ASF antibodies, making them suitable for ASFV serological diagnosis and epidemiological studies [48,49]. This discrepancy could be attributed to the difference between the two expression systems (*E. coli* vs. baculovirus), which affect antibody binding to the protein in iELISA [49], or modifications in the cloning and purification method used to acquire the recombinant antigens, which can also have an impact on antibody detection capability by altering test sensitivity. In addition, the difference may be due to the number and type of sample evaluated, i.e., whether the samples were collected from acutely infected animals prior to the development of the antibodies that are measurable by the ELISAs or from experimentally inoculated or field-infected domestic and wild pigs. For example, the analysis of sera collected from experimental and field ASF-positive samples compared with IPT as the reference test revealed differences in the sensitivity values ranging from 22.22% to 50% using OIE-ELISA, IDvet, Svanova, and Ingenesa ELISA kits. In fact, serum samples from domestic pigs experimentally infected with genotype II ASFV revealed that the IPT was more sensitive than the ELISA [48]. Furthermore, to be statistically acceptable, a test’s sensitivity and specificity values must be at least 1.5 (<1 indicates a useless test, and 2 indicates a perfect test) [37]. Our study resulted in 0.88 for sensitivity and 0.97 for specificity, giving us a sum of 1.85, indicating acceptable statistical support. Interestingly, during the validation process with a reference serum panel, the antibody detection capacity of the developed iELISA was observed in positive sera up to a dilution of 1:1000, indicating a high detection capacity of the iELISA.

Finally, the results of this study using the ASF-p72-truncated protein on iELISA for antibody detection showed nearly perfect agreement compared with the WOAH-recommended confirmatory IPT method (K = 0.872) and better compared with the kappa value of 0.34 to 0.64 reported with OIE-ELISA, IDvet, Svanova, and Ingenasa ELISAs, which showed moderate to substantial agreement between the IPT- and the ELISA-tested ASF-positive samples [48]. The inter-assay reproducibility of ASF-p72-truncated protein on iELISA was CV 10.4%, and the intra-assay reproducibility was CV 2.8%. These values demonstrate the precision and repeatability of the iELISA developed in this study because inter-assay percent CVs of less than 15 are generally acceptable, and intra-assay percent CVs should be less than 10%; these scores reflect the good performance of the assay. These values are in general agreement with the values presented in previous reports by Tighe et al., 2015 [34], who considered it important to ensure usable intra- and inter-assay reproducibility. Inter-assay CVs of <20%, and usually <10%, are a reasonable expectation of most ELISA-based systems, and intra-assay CVs of <15%, and usually <10%, should be expected, indicating good reproducibility.

As a result, the iELISA developed in this work could be suitable for application in epidemiological surveillance programs of infected pigs. Given the current scenario of emerging ASFV, the early detection of infected pigs is imperative for the timely implementation of control measures that mitigate the spread of the disease [19]. This study has the potential to establish a BSL-2 diagnostic laboratory through INIFAP-CENID-SAI-Mexico as part of the federal government. This could help address an ASF emergency in Mexico and support an early diagnosis with biotechnology tools. It could also serve as the basis for future development of other ASFV serological detection methods, such as pen-site lateral flow assays.

On the other hand, work carried out in our laboratory has established new generation techniques that have demonstrated the feasibility of producing various recombinant proteins in BL21 *E. coli* using the pET-SUMO vector. Despite not offering post-translational modifications, the conservation of antibody-producing epitopes indicates them as candidate antigens [27,28,29,30]. The production of recombinant proteins in pET-SUMO generated large quantities of functional viral antigens with excellent immunogenic and antigenic properties, regardless of the 6-His and SUMO tag modifications, with advantages in terms of protein solubility, stability, and purification [50]. The strong signals detected with Coomassie staining and Western blotting techniques indicate that the ASF-p72-truncated protein is efficiently expressed. This increases the possibility that the expressed ASF-p72-truncated protein preserves its intended activity and characteristics, improving diagnostic test sensitivity and specificity as well as enhancing adaptability to iELISA. This strategy was used to optimize the overproduction of recombinant protein utilizing commercially available goods at a competitive cost. In fact, the developed iELISA was optimized to use 30 ng/well of purified protein, in comparison to another reported indirect ELISA, where a concentration of purified protein (p30 and pB602L) exceeded that value by more than ten-fold per test (600 ng/well) [47]. In this study, a significant yield production of purified protein was obtained (231 μg/100 mL), showing high performance in the optimization of protein concentration and low cost for application in diagnostic systems such as iELISA. It is estimated that 70 diagnostic plates with a potential of 2940 double tests can be obtained from 100 mL of bacterial culture.

Additionally, in our investigation, we assessed the antigenicity of the ASF-p72-truncated protein form using a mouse model’s humoral immune response. An iELISA was utilized to assess antibody concentrations generated in CF1 mice inoculated with different immunostimulatory complexes. When the p72-truncated protein was mixed with an adjuvant (Matrix-M^TM^), a strong humoral response was induced in mice at day 14 post-inoculation, with an increase in levels at day 28. This efficient immune response has been reported with different recombinant antigens mixed with adjuvant matrix-M^TM^ [27,28,29,30], in which a lipid nanoparticle is widely used as an adjuvant to promote antigen recognition in immune system cells, enhancing antigenic folding and epitope recognition [51]. In contrast, when the mice were immunized with truncated ASF-p72 protein plus PBS, antibody detection started at 28 days post-inoculation, indicating a diminishing ability to generate an early humoral immune response. This could be associated with the fact that only a fragment of the ASF-p72 protein was expressed because it is well known that the ASF-p72 protein is considered a strong immunogen that can induce a high immune response in pigs and has been involved in the creation of subunit vaccines and understanding their immunogenic potential [4,13,52,53]. Therefore, it is proposed that the ASF-p72-truncated protein can be used as an immunogen if an adjuvant is added; however, further research and field trials are required.

## 5. Conclusions

In conclusion, according to our findings, the sequence of the truncated ASF-p72 recombinant protein has a high level of conservation when compared with sequences reported from recent outbreaks over the last ten years in affected countries. It was demonstrated to be a suitable ASF viral protein for successful expression in *E. coli* BL21 (DE3) from an insoluble phase (inclusion body). Thus, the proposed serological test provides a relevant and cost-effective tool for monitoring many samples and represents an ideal alternative for determining whether animals are sick or free of the disease. This technological development can be applied to future studies focusing on various biological and epidemiological challenges, making it useful for ASF surveillance in affected nations, including ASF-free American countries.

## Figures and Tables

**Table 1:** The frequency distribution of positive or negative sera was determined in the optical density (OD) ranges to determine the strong positive control serum (C++), the weak positive control serum (C+), and the negative control serum (C−). IPT: immunoperoxidase test; CV: coefficient of variation; SD: standard deviation.

$\bar{X}$ Strong-Positive ASF Serum (C++) IPT Value 1:1280	$\bar{X}$ Weak-Positive ASF Serum (C+) IPT Value 1:180	$\bar{X}$ Negative ASF Serum (C−) C−
1.143 ± 0.178 (1 SD)	0.506 ± 0.089 (1 SD)	0.147 ± 0.030 (1 SD)
1.143 ± 0.357 (2 SD)	0.506 ± 0.179 (2 SD)	0.147 ± 0.060 (2 SD)
Range C++ 0.786 to 1.50 OD	Range C+ 0.326 to 0.596 OD	Range C− 0.08 to 0.208 OD
CV 17%		CV 3%

$Inter-assay\;CV\%\;\left(n=10\right)=17.8\%\;CV\;of\;positive\;serum\;control+3\%\;CV\;of\;negative\;control/2=10.4\%.$

**Table 2:** Samples from reference panel sera (2019, 2020, 2021, and 2022) of the interlaboratory intercomparison serological assay tested by ASF commercial ASFV-blocking ELISA* INgezim^®^ PPA COMPAC 11.PPA.K3 (INGENASA), which uses a monoclonal antibody (MAb) specific to VP72 ASFV protein. “A”: IRTA-CReSA OIE Reference Laboratory at Barcelona, Spain. “B”: OIE Reference Laboratory “Laboratorio Central de Veterinaria”, Algete, Madrid, Spain. OD: optical density.

		Laboratory “A”		Laboratory “B”			
ID	Description	ELISA*-Ingezim PPA Compac Corta	ELISA*-Ingezim PPA Compac Larga	ELISA* Indirect % Bloqueo Compet	ELISA* Indirect Bloqueo Compet	OD of ASF-p72t Protein iELISA	ASF-p72t Protein iELISA
2019.1	Pig serum experimentally infected with PPA strain NH/68. Bleeding 30 dpi.	Neg/DUD	Pos	65.97	pos	0.883	Pos
2019.2	Pig serum (C2) infected and experimentally re-infected with ASFV (NHV/68 strain). Bleeding 63 dpi/28 dpi.	Pos	Pos	94.04	Pos	0.73	Pos
2019.8	Uninfected pork serum. Undiluted	Neg	Neg	0.27	Neg	0.12	Neg
2019.10	Uninfected pork serum. Undiluted	Neg	Neg	−2.07	Neg	0.179	Neg
2020.1	Sow Filtered Serum 3161/NEG) LCV Animal Facility	Neg	Neg	18.6	Neg	0.122	Neg
2020.3	Sow Filtered Serum 3161/NEG) LCV Animal Facility	Neg	Neg	9.9	Neg	0.168	Neg
2020.4	27 February 2020 and lyophilized on 6 January 2020. Proceeds from infection (and re-infection) experimentally with VPPA strain NHV/68. Indentation 63 dpi -28 dpi.	Pos	Pos	102.8	Pos	0.558	Pos
2020.5	7 April 2019 and lyophilized on 7 September 2019. Proceeds of an infection (and re-infection) experimentally with the VPPA strain NHV/68. Bleeding 63 dpi -28 dpi.	Pos	Pos	97.9	Pos	0.597	Pos
2020.6	27 February 2020 and lyophilized on 1 June 2020. Proceeds of an infection (and re-infection) experimentally with VPPA strain NHV/68. Bleeding 63 dpi -28 dpi.	DUD	Pos	77.7	Pos	0.506	Pos
2020.8	27 February 2020 and lyophilized on 1 June 2020. Proceeds from infection (and re-infection) experimentally with VPPA strain NHV/68. Bleeding 63 dpi -28 dpi.	NEG/DUD	Pos	63.2	Pos	0.337	Pos
2020.9	7 April 2019 and lyophilized on 7 September 2019. It is from an experimentally infected with VPPA strain NHV/68. Bleeding 30 dpi.	Pos	Pos	96.9	Pos	0.614	Pos
2021.1	7 May 2021 and lyophilized on 19 July 2021. It is from an infection (and re-infection) experimentally with VPPA strain NHV/68. Bleeding 63 dpi -28 dpi.	Pos	Pos	89.9	Pos	0.862	Pos
2021.2	27 February 2020 and lyophilized on 2 June 2020. It is from an infection (and re-infection) experimentally with VPPA strain NHV/68. Bleeding 63 dpi -28 dpi.	Neg/DUD	Pos	75.2	Pos	0.97	Pos
2021.3	5 July 2021 and lyophilized on 19 July 2021. It is from an infection (and re-infection) experimentally with the VPPA strain NHV/68. Bleeding 63 dpi -28 dpi.	Pos	Pos	93.4	Pos	0.543	Pos
2021.5	27 February 2020 and lyophilized on 2 June 2020. Proceeds from infection (and re-infection) experimentally with VPPA strain NHV/68. Bleeding 63 dpi -28 dpi.	Pos	Pos	97.3	Pos	0.562	Pos
2021.7	27 June 2020 and lyophilized on 5 June 2020. Proceeds from infection (and re-infection) experimentally with VPPA strain NHV/68. Bleeding 30 dpi.	Pos	Pos	99.2	Pos	0.476	Pos
2022.1	Uninfected pork serum. Undiluted.	Neg	Neg	0	Neg	0.147	Neg
2022.5	7 April 2022 and lyophilized on 11 May 2022. It is derived from an experimental infection (and re-infection) with the VPPA strain NHV/68.	Pos	Pos	99	Pos	0.938	Pos

**Table 3:** The 2 × 2 contingency table. Sensitivity and specificity analysis: a, the number of true positives; b, the number of false positives; **c**, the number of false negatives; and d, the number of true negatives. The calculated concordance index (index κ) of iELISA was κ = 0.872, indicating a nearly perfect agreement considering immunoperoxidase (IPT) as the “gold standard”.

	**IPT-Positive Serum**	IPT-Negative Serum	Total
Positive serums	a (22)	b (1)	r = a + b (23)
Negative serums	c (3)	d (43)	s = c + d (46)
Total	t = a + b (25)	u = b + d (44)	N = a + b + c + d (69)
Sensitivity	$\%sensitivity=\left[\frac{a(22)}{a\left(22\right)+c(3)}\right]*100=88\%$		
Specificity	$\%specificity=\left[\frac{d(43)}{b\left(1\right)+d(43)}\right]*100=97.72\%$		
Kappa index	$Po=\frac{\left(22+43\right)}{69}=0.942;\;Pe=\frac{\left(23\times 25\right)+(46\times 44)}{69^{2}};\;K=0.942-\frac{0.545}{1-0.545}=0.872$		

Abbreviation: IPT, immunoperoxidase.

## Data Availability

The original contributions presented in this study are included in the article/Supplementary Materials. Further inquiries can be directed to the corresponding author.

## Supplementary Materials

The following supporting information can be downloaded at https://www.mdpi.com/article/10.3390/pathogens14060542/s1, Table S1: Optical density of serological results of 22/23 reference positive serum samples evaluated by iELISA ASF-p72-truncated protein and INgezim^®^ PPA ELISA kit. nd: not determined; Table S2: Optical density of serological results of 43/46 reference negative serum samples evaluated by iELISA ASF-p72-truncated protein and calculated coefficient of variation percent (% CV) as the intra-assay evaluation % CV = 2.8.

## Abbreviations

The following abbreviations are used in this manuscript:

ASF	African swine fever
ASFV	African swine fever virus
CReSA	Centre de Recerca en Sanitat Animal
CV	coefficient of variation
DP	double positive
ELISA	enzyme-linked immunosorbent assay
EURL	European Union Reference Laboratory
HRP	horseradish peroxidase
IB	immunoblotting
iELISA	indirect enzyme-linked immunosorbent assay
IIF	indirect immunofluorescence
IMAC	immobilized metal affinity chromatography
IPT	indirect immunoperoxidase test
IPTG	isopropyl β-D-1-thiogalactopyranoside
IRTA	Institute for Research and Technology in Food and Agriculture
MAb	monoclonal antibody
OIE	Organization for Animal Health
ORF	open reading frame
SC	subcutaneous
SD	standard deviations
TMB	3,3′,5,5′-tetramethylbenzidine
κ	kappa
